# Supplementary material for: FicD sensitizes cellular response to glucose fluctuations in mouse embryonic fibroblasts
Source: Proc Natl Acad Sci U S A. 2024 Sep 11;121(38):e2400781121. doi: 10.1073/pnas.2400781121 (PMC11420183; doi:10.1073/pnas.2400781121)
Supplement: Supplementary file 1 — Appendix 01 (PDF) [file pnas.2400781121.sapp.pdf]

Supporting Information for

FicD Sensitizes Cellular Response to Glucose Fluctuations in Mouse Embryonic Fibroblasts

Burak Gulen[1,2], Lisa N. Kinch[1,2], Aubrie Blevins[1], Kelly A. Servage[1,2], Nathan M. Stewart[1,2], Hillery F. Gray[1,2], Amanda K. Casey[1,2], and Kim Orth[1,2,3,*]

*Corresponding author: Kim Orth[1]
Email: kim.orth@utsouthwestern.edu

This PDF file includes:

Supplementary Information Text
Figures S1 to S6
Supplemental Table S1

Other supporting materials for this manuscript include the following:

Datasets S1 to S2

Supplementary Information Text

SI Materials and Methods

Reagents and general remarks

All the reagents were purchased from Thermo Fisher Scientific and Sigma unless otherwise stated and were of appropriate grade. Antibodies were purchased from Abcam, Cell Signaling, and Thermo Fisher Scientific except the monoclonal α-AMP antibody which is a generous gift of Aymelt Itzen (University Medical Center Hamburg Eppendorf, Germany). Wild-type and IRE1$^{-/-}$ MEFs were kind gifts from Jenna Jewell and Fumihiko Urano, respectively. PERK$^{-/-}$ MEFs were purchased from ATCC (ATCC no. CRL-2976). Unless otherwise stated, all the qPCR experiments were repeated at least three times and the western blot experiments repeated at least twice, by using distinct samples.

Isolation and immortalization of MEFs

Isolation: *FicD*$^{F/F}$ and *FicD*$^{-/-}$ mouse embryos (E13.5) were cleaned from placenta and membrane in PBS in a 10 cm petri dish under dissection microscope. Embryos were broken by sucking and exhausting 5 times with 2.5 mL syringe with 18G needle. Each broken embryo placed into 10 cm dish in 10 mL complete medium: High-Glucose DMEM medium (Sigma, D5796) supplemented with 10% FBS (Sigma, F2442), 1 mM sodium pyruvate (Sigma), 1x penicillin/streptomycin-L-glutamine (Sigma). Cells were grown in 5% CO_2 at 37 °C for 2 days, large bones and unbroken pieces were removed by sedimentation, and split 1/3 ratio. Splitting and sedimentation were repeated every 2-3 days total 3 times and cells were collected by centrifugation and frozen with 10% DMSO/complete medium by slow cooling (20 °C 1h to 80 °C) in cryotubes.
Immortalization: Thawed MEFs were split into 6 well plates by ¼ and 1/6 dilutions and grown overnight in 5% CO_2 at 37 °C. Approximately 25% confluent wells were transfected with 2 μg plasmid vector expressing SV40 antigen using Fugene Transfection Reagent (Promega) as described by manufacturer. MEFs were incubated overnight in 5% CO_2 at 37 °C and medium was exchanged. Two days after transfection, transfected MEFs were split into 10cm dish. MEFs were split to ultimately 1/100,000 fold dilution by observing the confluence over the course of 3-4 passages to eliminate non-transformed cells. Immortalized MEFs were frozen by slow cooling and stored in liquid nitrogen.

Cell culture

MEFs were grown to 80–90% confluency in standard 10cm cell culture dish (VWR) and cultured in complete medium: High-Glucose DMEM medium (Sigma, D5796) supplemented with 10% FBS (Sigma, F2442), 1 mM sodium pyruvate (Sigma), 1x penicillin/streptomycin-L-glutamine (Sigma). MEFs were incubated at 5% CO2 at 37 °C. Pharmacological treatment of MEFs were carried out in complete medium supplemented with 0.1-10 μM thapsigargin in DMSO or 5mM DTT in PBS or 100 μg/mL CHX. Glucose starvation was carried out in glucose-free medium: Glucose-free DMEM (Thermo Fisher, 11-966-025) supplemented with 10% FBS (Sigma, F2442), 1x penicillin/streptomycin-L-glutamine (Sigma).

Cell Harvesting and Lysis

MEFs were grown in 6-well plates or 10cm dishes at 5% CO_2 at 37 °C to 80% confluence. Treated MEFs were either harvested by trypsinization or transferred to ice and harvested by scraping and transferred to 1.5 mL reaction tubes on ice. Medium was aspirated after centrifugation at 1000xg for 5 min at 4 °C and cell pellet was washed with ice cold 1x PBS. MEFs were then frozen in liquid nitrogen and stored at 80 °C. To prepare cells for Western blotting, the cell pellet was resuspended in lysis buffer (50 mM Tris pH 7.4, 150 mM NaCl, 0.2% Triton, 1x Roche Complete EDTA free protease inhibitor cocktail, 1x Roche PhosSTOP, and approximately 0.5units/uL Novagen Benzonase nuclease) and chilled on ice for 5-10 minutes. Lysed MEFs were then resuspended in RIPA buffer (50mM Tris pH 7.4, 0.5% NP40, 0.5% DOC, 0.2% SDS, 1X Roche EDTA free protease inhibitor cocktail, 1X Roche PhosStop, 1mM DTT) before centrifugation at full speed for 10 min on a bench top Eppendorf 5424 R centrifuge at 4 °C. Clarified supernatants were transferred to fresh Eppendorf tube tubes. Lysate protein concentrations were determined by Pierce BCA Protein Assay (Thermo Scientific) and normalized in RIPA buffer before the addition of 0.5-1 volumes of 5X SDS sample buffer (250mM Tris pH 6.8, 10% SDS, 100mM DTT, 50% glycerol, 0.25% Bromophenol Blue). For mRNA isolation, frozen MEFs were lysed directly with QIAshredder columns (Qiagen).

Protein Synthesis Assay

MEFs were cultured and subjected to glucose starvation and refeeding as previously described. Thirty minutes prior to cell harvesting, the culture medium was replaced with a pre-warmed medium containing 1 μM Puromycin (Thermo Scientific), appropriate for the specific condition being tested (either regular or glucose-free medium). The cells were then incubated under 5% CO_2 at 37°C for the final 30 minutes, followed by harvesting and lysis as previously described. Western blot analysis was conducted according to the protocol detailed in the subsequent section.

Western-blotting

Normalized lysate samples were resolved on 8%, 10%, or 12% SDS-PAGE gels using Mini-PROTEAN® Tetra Cell (Bio-Rad) and transferred to Millipore Immobilon®-P 0.45uM Pore size PVDF Membrane for 10 min at 1.5 Amps with an Invitrogen PowerBlotter.The membranes completely dried at 37C for 10 minutes, rewetted in 100% MeOH, rinsed in 1X TTBS, and rocked in blocking buffer (5% BSA, 5% milk, or 1x Roti-Block (Carl Roth) in 1X TTBS) for 1 h at room temperature. Primary antibodies were diluted in appropriate blocking buffer before incubation with membranes overnight at 4 °C. Antibody dilutions are as follows: 1:15,000 α-AMPylation (17G6) (gift from Aymelt Itzen), 1:10,000 α-GRP78 (Abcam, ab21685), 1:1,000 α-phospho-eIF2α-Ser51 (Abcam, ab32157), 1:1,000 α-eIF2α (Cell Signaling, 9722), α-puromycin (Developmental Studies Hybridoma Bank, PMY-2A4), 1:30,000 α-Actin (Sigma A228), 1:1,000 α-CHOP (Cell Signaling, #2895), 1:1,000 α-IRE1 (Cell Signaling, #3294), and 1:1,000 α-PERK (Cell Signaling, #3192). Membranes were then washed in 1X TTBS before incubation with either 1:5,000 donkey α-rabbit-HRP (Amersham, NA934) or 1:10,000goat α-mouse-HRP (Abcam, ab205719) for 1 hour at room temperature. The membranes were then washed in 1X TTBS, developed with Advansta WesternBright

ECL Spray or ThermoScientific SuperSignal™ West Femto Maximum Sensitivity Substrate and imaged with a ChemiDoc Imaging System (Bio-Rad).

RNA isolation, RT-qPCR, and RNA-seq

Total RNA from MEFs were isolated by RNeasy Plus Mini Kit (Qiagen) as described by the manufacturer after lysing the MEFs by QIAshredder columns (Qiagen). RNA concentrations were measured by nanodrop (Thermo Scientific). Normalized RNA samples were digested with DNase I (Thermo Scientific) in 20 µL reaction (2 µg total RNA) for 30 min at 37 °C. DNase reaction was stopped by adding 2 µL of EDTA (50mM) at 65 °C for 10 min. DNase treated RNA was reverse transcribed by qScript cDNA synthesis kit (Quanta Bio) in a 40 µL reaction as descried by the manufacturer. Quantitative PCR was performed with 10 ng cDNA by using PowerTrack SYBR Green Master Mix (Thermo Scientific) in either 96 well plates or 384 well plates using either CFX 96 (Bio-Rad) or CFX Opus 384 (Bio-Rad) respectively. U36B4 (NM_007475) was used as the reference mRNA. Results were analyzed by CFX Maestro Software (Bio-Rad) and plotted by Prism 9 (Graphpad). Primers used for the genes analyzed in this study (U36B4 forward primer: 5' cgtcctcgttggagtgaca 3', reverse primer: 5' cggtgcgtcagggattg 3'; ATF3 forward primer: 5' tggagatgtcagtcaccaagtct 3', reverse primer: 5' gcagcagcaattttatttctttct 3'; ATF4 forward primer: 5' actctaatccctccatgtgtaaagg 3', reverse primer: 5' caggtaggactctgggctcat 3'; CHOP forward primer: 5' ccagaaggaagtgcatcttca 3', reverse primer: 5' actgcacgtggaccaggtt 3'; sXBP1 forward primer: 5'ctgagtccgcagcaggt 3', reverse primer: 5' tgtcagagtccatgggaaga 3'; BiP forward primer: 5' caaggattgaaattgagtccttctt 3', reverse primer: 5' ggtccatgttcagctcttcaaa 3'; FicD forward primer: 5' gtagacgcactgaatgagttcg 3', reverse primer: 5' tggtgtataagtagtcagcctgg 3'. For RNA-seq experiment, isolated RNAs are first passed the quality control then sequenced by Novogene Corporation Inc. and raw data is mapped with CLC Genomics Workbench software (version 9.5, CLC Bio, Aarhus, Denmark).

Analysis of RNA-seq

Fastq reads corresponding to each of four treatments (unstressed, glucose starved, 2-hour refed, and 4-hour refed) applied to *FicD*$^{F/F}$ and *FicD*$^{-/-}$ MEFs (all conditions in triplicate) were mapped to the mouse reference genome, and statistical analysis was performed using CLC Genomics Workbench software (version 9.5, CLC Bio, Aarhus, Denmark). Total counts and CPM for each mouse gene were generated for all conditions (8 conditions in triplicate, 24 samples). Principal Component Analysis (PCA) was calculated for all samples with ClustVis (*60*) using Log2 CPM counts with unit variance scaling and row centering. Principal components were calculated using Singular Value Decomposition (SVD) with imputation.

Total counts mapped for each sample were used to calculate differential gene expression with four methods: EdgeR, DESeq2, limma, and NOISeq using the Integrative Differential Expression Analysis for Multiple EXperiments (IDEAMEX) server with chosen parameters (LogFC=0.75, FDR=0.05, and CPM=1) without batch effects (*61*). Differentially expressed genes (DEGs) were identified for various pairwise conditions (**Fig. 2C**). Differential expression results were integrated to observe the consistency of DEGs identified by each method **(Fig. S3A** and **B**). Ultimately, EdgeR (*62*) DEGs were chosen for subsequent GO term enrichment.

For GO term enrichment analysis, Ensemble IDs for upregulated EdgeR DEGs were submitted to the G:profiler server (*63*) limiting the statistical domain scope to annotated genes, using the G:SCS threshold to calculate adjusted P-values with a cutoff below 0.01, limiting pathway size to between 10 and 250 terms, and excluding electronic GO annotations. We removed redundant terms with identical lists of genes, keeping the top term ranked by the lowest P-value. We report the enriched GO biological process terms for upregulated DEGs from metabolic stress comparison of $Fic^{FL/FL}$ starved/$FicD^{F/F}$ unstressed MEFs and for enriched GO molecular function terms for upregulated DEGs from genotype comparison of $FicD^{-/-}$ with $FicD^{F/F}$ MEFs.

PCA and heat maps were generated for mouse DEGs (identified an any of the RNA-seq comparisons from Fig3C, as well as DEGs from any of the conditions compared to the respective unstressed state) with GO terms related to unfolded protein response (UPR) as defined in UniProt (*64*). Genes were clustered in the heatmap by hierarchical clustering (Euclidean distance with Ward method) using clustvis with the same parameters as used for PCA (*60*).

Preparation of Secretomes

After overnight incubation in serum-containing media with or without glucose, the culture media of MEFs were replaced with serum-free media with or without glucose. Following an additional incubation period of 2-4-hours, the supernatants from the MEFs were collected into 1.5 mL tubes placed on ice. To remove cells and debris, the supernatants were centrifuged for 15 minutes at 3200 x g at 4 °C. Subsequently, the supernatants were filtered through a 0.22µm filter into fresh microcentrifuge tubes. To the filtered supernatants, a final concentration of 150 µg/ml sodium deoxycholate was added and incubated for 15 minutes on ice. Furthermore, a final concentration of 8% (v/v) trichloroacetic acid was added to each sample, followed by an overnight incubation at 4 °C. The precipitated proteins were resuspended and transferred to fresh microcentrifuge tubes that had been pre-rinsed with methanol to remove collagen contamination. These suspensions were then subjected to centrifugation for 1 hour at 27,000 x g at 4 °C. Afterward, the supernatants were discarded, and the pellets were washes twice with 1.5 ml of pre-cooled 100% acetone, with each wash involving centrifugation for 40 minutes at 27,000 x g at 4 °C. Following the second wash, the pellets were air-dried for 10 minutes and then resuspended in 1 ml of 10 mM Tris-HCl at pH 8.0. The resuspended samples were transferred to fresh microcentrifuge tubes in preparation for tryptic digestion.

Tandem Mass Spectrometry

Secreted protein samples were reduced with 10mM DTT for 1 hr at 56°C and alkylated with 50mM iodoacetamide for 45 min at room temperature in the dark. Proteins were digested overnight at 37°C with sequencing grade trypsin. Resulting peptides were then de-salted via solid phase extraction (SPE) prior to analysis. LC-MS/MS experiments were performed on a Thermo Scientific EASY-nLC 1200 liquid chromatography system coupled to a Thermo Scientific Orbitrap Fusion Lumos mass spectrometer. To generate MS/MS spectra, MS1 spectra were first acquired in the Orbitrap mass analyzer (resolution 120,000). Peptide precursor ions were isolated and fragmented using high-energy collision-induced dissociation (HCD). The resulting MS/MS fragmentation

spectra were acquired in the ion trap. Label-free quantitative searches were performed using Proteome Discoverer 2.2 software (Thermo Scientific). Samples were searched against all reviewed entries in the Mouse UniProt protein database. Searches included the following modifications: carbamidomethylation of cysteine residues (+57.021 Da), oxidation of methionine (+15.995 Da), and acetylation of peptide N-termini (+42.011 Da). Precursor and product ion mass tolerances were set to 10 ppm and 0.6 Da, respectively. Peptide spectral matches were adjusted to a 1% false discovery rate (FDR) and proteins were filtered to a 5% FDR. All samples were run in biological triplicate. Data is reported in **Supplemental Dataset S2**.

Analysis of Secretomes

Proteins were mapped from the following treatment and genotype comparisons: starved *FicD*$^{F/F}$ / unstressed *FicD*$^{F/F}$, starved *FicD*$^{-/-}$ / unstressed *FicD*$^{-/-}$, unstressed *FicD*$^{-/-}$ / unstressed *FicD*$^{F/F}$, and starved *FicD*$^{-/-}$ / starved *FicD*$^{F/F}$. Upregulated proteins were defined as having an abundance ratio fold change = >1.5 and p-value < 0.05, as well as proteins with missing values that were identified in at least 2 reps of the first condition (i.e. starved *FicD*$^{F/F}$ from starved *FicD*$^{F/F}$ / unstressed *FicD*$^{F/F}$) but 0 reps of the second (i.e. unstressed *FicD*$^{F/F}$ from starved *FicD*$^{F/F}$ / unstressed *FicD*$^{F/F}$). Proteins with low combined FDR confidence (<0.05) were excluded. The same cutoffs were used to identify downregulated proteins (abundance ratio = <1.5 and p-value < 0.05, as well as proteins with missing values that were identified in at least 2 reps the second condition but 0 reps of the first condition). Secreted proteins were identified from UniProt annotations for GO cellular component terms that include “extracellular” or for subcellular location terms that include “secreted”.

Fig. S1.

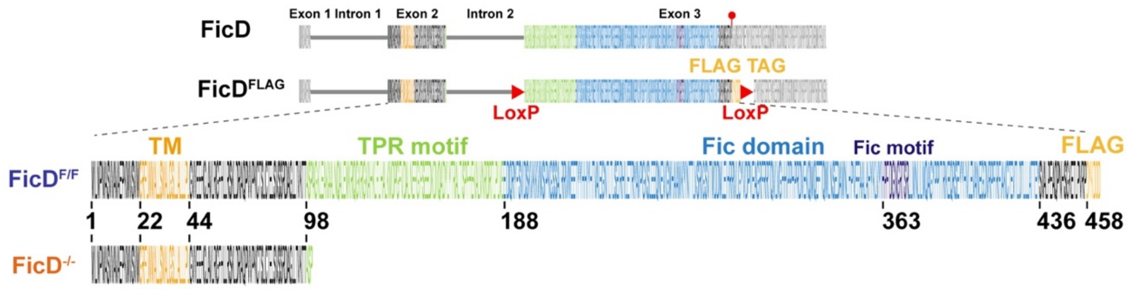

Figure S1. FicD knockout mouse embryonic fibroblasts (MEFs). FicD-FLAG and FicD knockout MEFs: *FicD*$^{F/F}$ and *FicD*$^{-/-}$. Insertion of FLAG tag to wild type MEFs and deletion of Fic domain and TPR motif from LoxP flanked *FicD*$^{F/F}$ MEFs is shown.

Fig. S2.

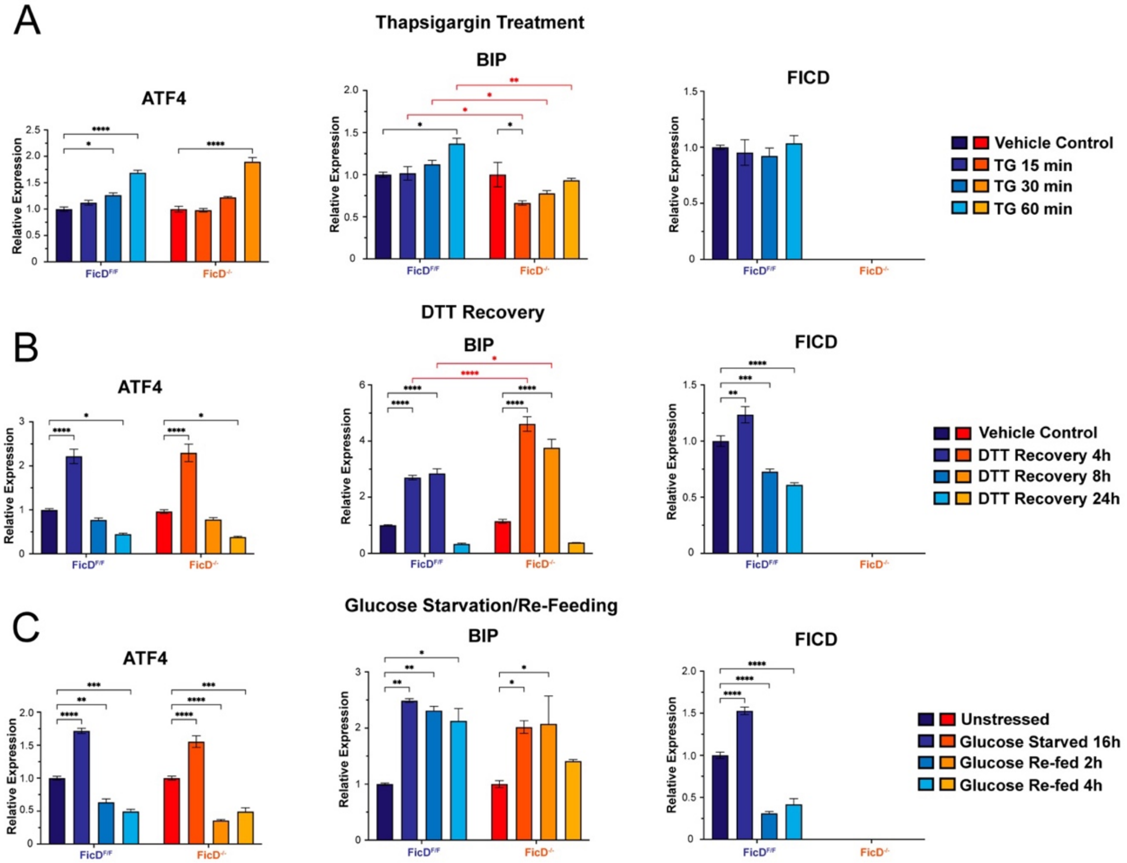

Figure S2. Thapsigargin (TG), DTT, and Glucose Starvation induced ER stress of *FicD*$^{F/F}$ and *FicD*$^{-/-}$ MEFs. RT-qPCR showing relative expression levels of UPR marker genes ATF4, BiP, and FicD upon **A)** treatment of MEFs with Thapsigargin (1µM), or **B)** recovery of MEFs from 1h DTT (5mM) exposure, or **C)** glucose starvation (for 16h) and re-feeding of MEFs for indicated time points. Error bars represent the standard deviation of 3 biologically independent repeat of the experiment with 4 technical replicates. Genotypes are demonstrated by shades of blue for *FicD*$^{F/F}$ and shades of orange for *FicD*$^{-/-}$ MEFs. Two-way ANOVA with Tukey multiple comparison test is applied to determine the significance. P values: 0.1234 (ns), 0.0332 (*), 0.0021 (**), <0.0001 (****). Non-significant comparisons are not shown for clarity.

Fig. S3

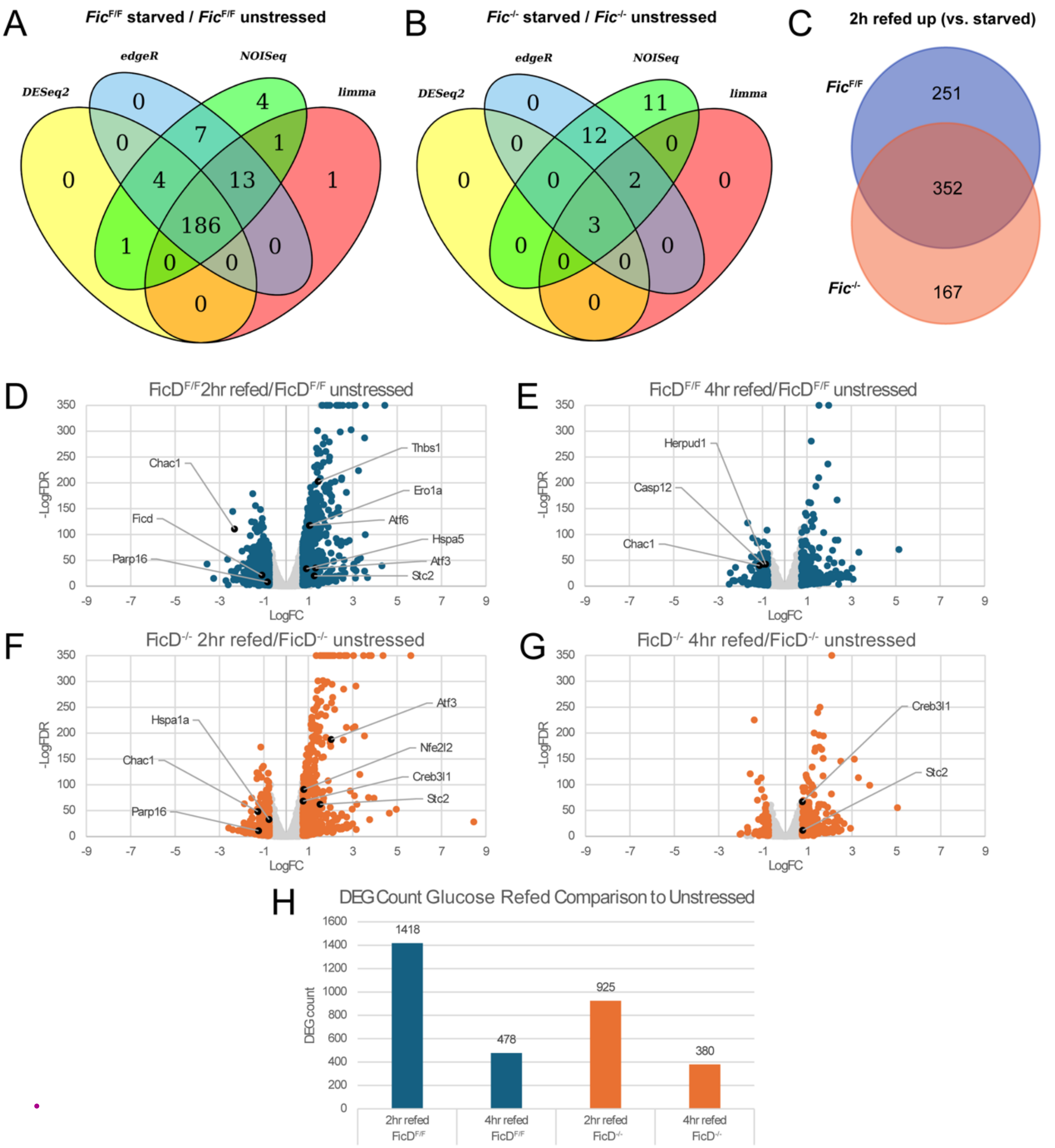

Figure S3. A-B) Venn diagrams compare different Rna-seq methods for identifying DEGs for **A)** *FicD*$^{F/F}$ starved / *FicD*$^{F/F}$ unstressed MEFs and B) *FicD*$^{-/-}$ starved / *FicD*$^{-/-}$ unstressed MEFs. **C)** Venn diagram comparing upregulated genes in both genotypes for 2h refed / starved MEFs. **D-G**) Volcano plots of LogFC against -LogFDR (with maximum of 350 assigned to genes with FDR 0) for indicated comparison conditions (plot titles), FicDF/F (blue) and FicD$^{-/-}$ (orange). UPR genes are labeled. **H**) Total DEG counts for indicated conditions.

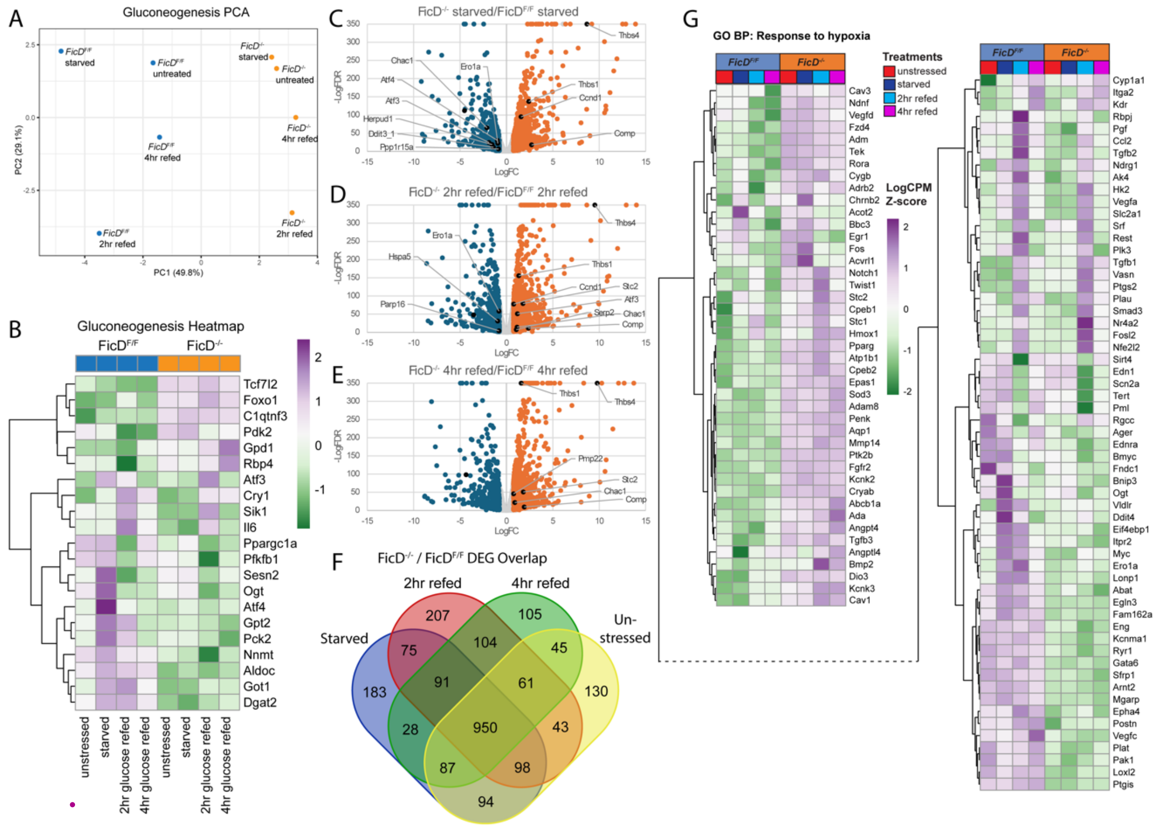

Figure S4. A) PCA plot and **B)** heatmap (colored by Log2CPM Z-score) depict significant DEGs under any condition with Go term including “gluconeogenesis”. Genes are clustered Euclidean distance and Ward linkage. **C-E**: Volcano plots of LogFC against -LogFDR (with maximim of 350 assigned to genes with FDR 0) for indicated comparison conditions (plot titles), up in FicD$^{F/F}$ (blue) and up in FicD$^{-/-}$ (orange). UPR genes are labeled. **F**) Venn diagram depicting DEG overlaps comparing FicD$^{-/-}$ to FicD$^{F/F}$ for the indicated conditions. **G)** Heatmap clustering Log2CPM (Z-score) of differentially expressed genes under any condition with GO BP term “response to hypoxia”.

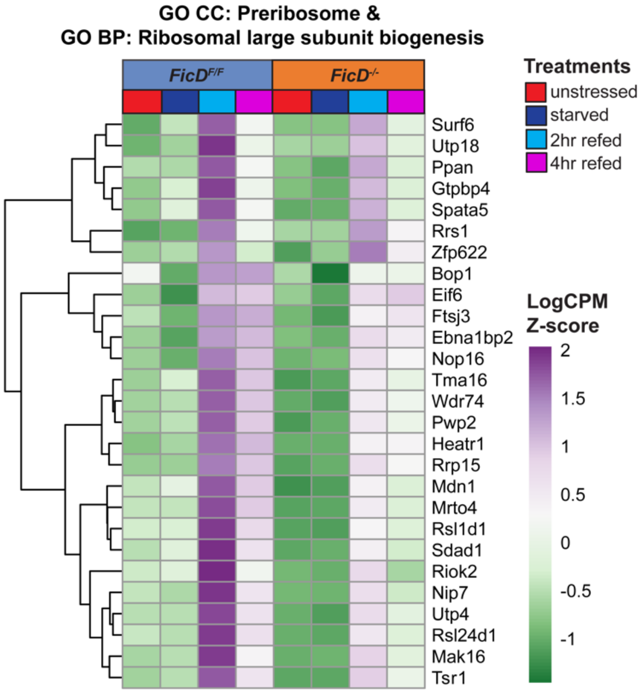

Figure S5. Heatmap (colored by Log2CPM Z-score) depicts significant DEGs under any condition with GO CC term including "Preribosome" and GO BP term including "Ribosomal large subunit biogenesis". Genes are clustered Euclidean distance and Ward linkage.

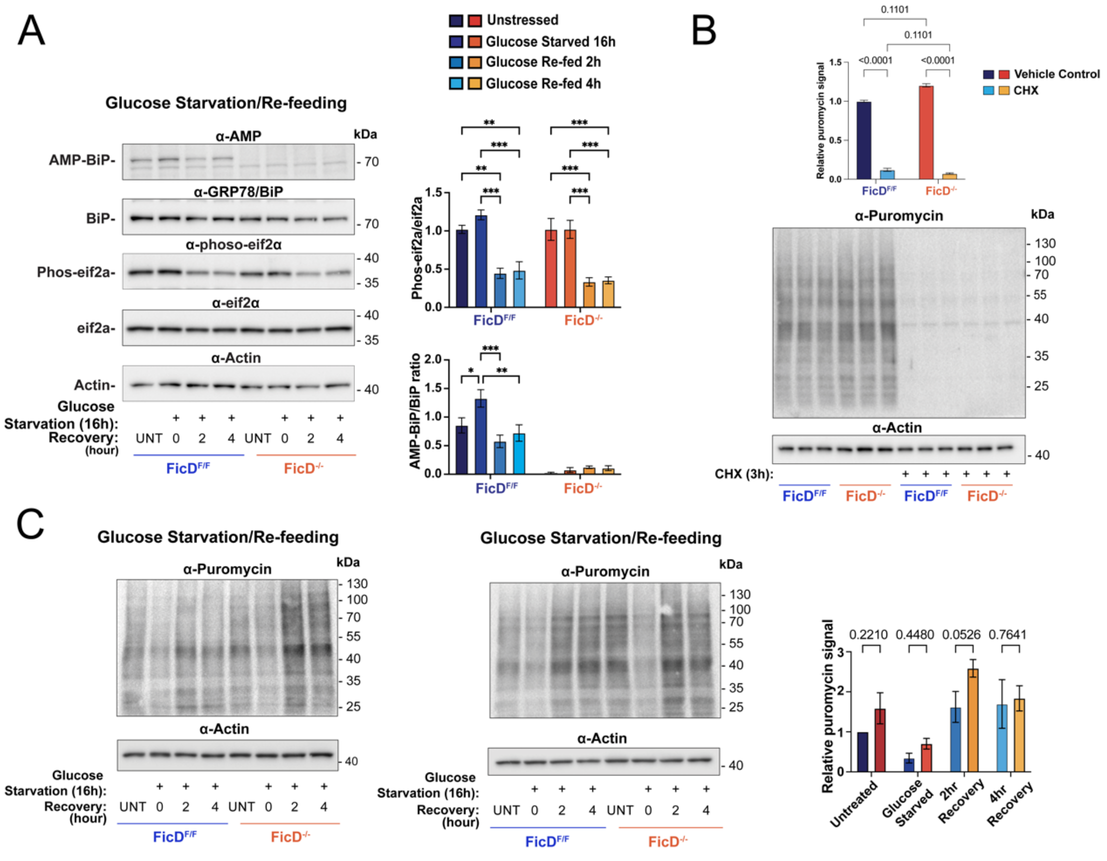

Figure S6. Nascent protein synthesis during glucose starvation and refeeding A) AMPylation and eIF2a phosphorylation of *FicD*$^{F/F}$ and *FicD*$^{-/-}$ MEFs during glucose starvation and refeeding. Bar graphs indicates the quantification of the rations of phospho-eIF2a/eIF2a and AMP-BiP/BiP. B) Cycloheximide control for puromycin treatment. Bar graph shows the quantification of western blot signals. C) Puromycin treatment assay during glucose starvation and refeeding. Two separate blots are shown. Bar graph indicates the quantification of western blot signals. Two-way ANOVA with Tukey multiple comparison test is applied to determine the significance. P values: 0.1234 (ns), 0.0332 (*), 0.0021 (**), <0.0001 (****). Non-significant comparisons are not shown for clarity.

Dataset S1 -Supplemental_Dataset_S1 (separate file)

Dataset S2 -Supplemental_Dataset_S2 (separate file)
